# Experimental hybridization and chromosome pairing in *Kosteletzkya* (Malvaceae, Malvoideae, Hibisceae), and possible implications for phylogeny and phytogeography in the genus

**DOI:** 10.3897/CompCytogen.v7i1.4542

**Published:** 2013-04-17

**Authors:** Orland J. Blanchard, Jr.

**Affiliations:** 1 University of Florida Herbarium, Florida Museum of Natural History, 379 Dickinson Hall, P. O. Box 110575, Gainesville, Florida 32611-0575, U.S.A.

**Keywords:** *Kosteletzkya*, Hibisceae, Malvoideae, Malvaceae, hybridization, chromosomes, phytogeography, phylogeny

## Abstract

*Kosteletzkya* C. Presl, 1835 (Malvaceae, Malvoideae, Hibisceae) includes 17 species, all but two of which are about evenly distributed between Africa and the northern Neotropics. Fifteen of the species were brought into cultivation and used in a hybridization program in an attempt to shed light on evolutionary and phytogeographic relationships in the genus. Chromosome pairing (x = 19) at meiosis was examined in 51 of the 56 interspecific hybrids that were produced, and the seven New World species, all diploids, were found to exhibit nearly complete pairing among themselves, indicating that they share a genome. By contrast the three African diploids showed low levels of chromosome pairing in crosses among themselves, leading to the recognition here of three distinct genomes, newly designated A, B and G. The African B-genome diploid, *Kosteletzkya buettneri* Gürke, 1889, was found to share its genome with the New World species. Four other African species are known to be tetraploids and a fifth, a hexaploid. The results of chromosome pairing in hybrids among all of the African species at all ploidy levels, plus the discovery of a spontaneously tetraploidized experimental intergenomic African diploid hybrid, suggest that three of the four tetraploids and the single hexaploid might all be allopolyploids built on the three known extant genomes. The fourth tetraploid paired poorly or moderately with these three genomes. Results are consistent with the hypothesis that *Kosteletzkya* arose in Africa, radiated at the diploid level, underwent natural interspecific hybridization, produced two tiers of allopolyploids, and at some more recent time dispersed a B-genome diploid to the New World where it underwent another radiation at the diploid level. Structural features of the fruits suggest adaptations for passive distribution by animals, potentially over long distances.

## Introduction

*Kosteletzkya* C. Presl, 1835 (Malvaceae, Malvoideae, Hibisceae) comprises 17 species that, with two exceptions, are about evenly divided between Africa (eight species) and the northern New-World tropics (seven species; Blanchard 2012). One of the exceptions, *Kosteletzkya pentacarpos* (Linnaeus, 1753) Ledebour, 1841, is found primarily extra-tropically along the eastern and Gulf coasts of the United States, with a few probably introduced populations in Eurasia (Blanchard 2012); the other exception, *Kosteletzkya batacensis* (Blanco, 1837) Fernández-Villar, 1880, is found only on the island of Luzon in the Philippines (Borssum-Waalkes 1966). At present, *Kosteletzkya* sits awkwardly within the paraphyletic genus *Hibiscus* Linnaeus, 1753 along with *Pavonia* Cavanilles, 1786, *Abelmoschus* Medikus, 1787,* Talipariti* Fryxell, 2001,* Wercklea* Pittier & Standley, 1916, and several other, mostly smaller genera (Pfeil and Crisp 2005), but *Kosteletzkya* itself is well circumscribed (Blanchard in Verdcourt and Mwachala 2009). Structurally the genus is distinctive among the Hibisceae in that its 5-valved, 5-angled or -winged capsules contain a single seed per locule, and the valves themselves ultimately separate both from one another and from the fruiting axis. This characteristic of fruit disintegration, along with other features, excludes several endemic Madagascan species that are generally placed in *Kosteletzkya*, but which clearly belong elsewhere. Recent DNA evidence supports this interpretation (Koopman and Baum 2008).

**Figure 1.**
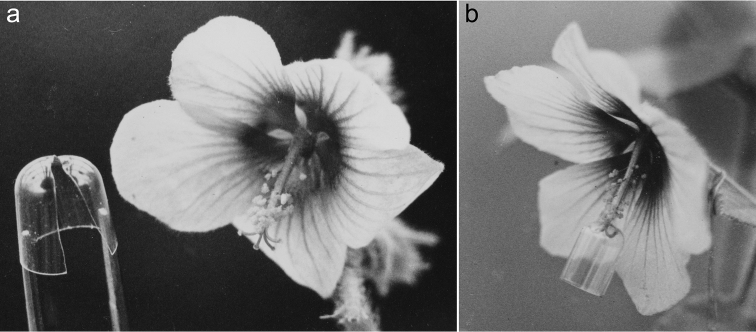
Simple gelatin-capsule device for preventing self-pollination in experimentally manipulated flowers of *Kosteletzkya*. **a** Perforated capsule-half spread with forceps in preparation for placement around the base of the style-branches of a flower of *Kosteletzkya begoniifolia* (76-1) **b** Capsule-half closed and in place between the base of the style-branches and the pollen mass. Note the recurved styles pressing their stigmas against the inside of the capsule-half.

The species of *Kosteletzkya* are mostly herbaceous perennials that bear small to medium-sized Hibiscus-like flowers (Figs 1, 6A–P) that usually last for a single day. Indigenous uses have been reported for several of the species (Chevalier 1940, Iljin 1949, Morton 1981, Anokbonggo et al. 1990, Burkill 1997), but only the temperate *Kosteletzkya pentacarpos* has received much attention for its more general economic potential (see Halchak et al. 2011). On account of its salt tolerance (Somers 1978, Grant and Somers 1981, Gallagher 1985, Blits et al. 1993, Poljakoff-Mayber et al. 1994) the plant can be grown as a crop on otherwise non-arable soil, and this has led in turn to studies that have identified *Kosteletzkya pentacarpos* as a potential commercial fiber source and have also shown that the seeds may be harvested as potential sources of biodiesel fuel and animal feed (Nekrasova and Pankova 1949, Islam et al. 1982, Ruan et al. 2008b). The same species has also found minor commercial use in the horticultural trade, especially for native-plant gardens.

**Figure 2.**
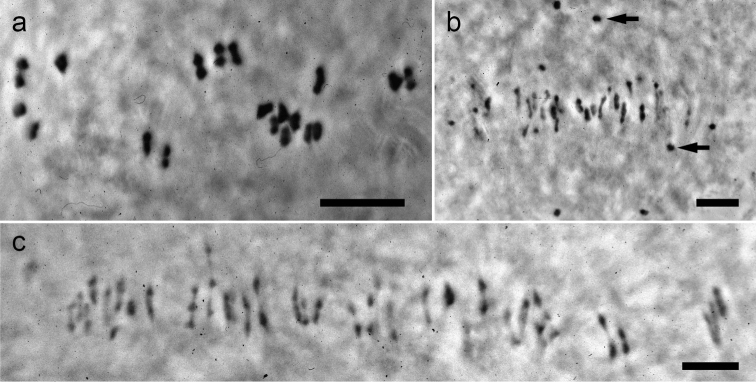
Phase-contrast photographs of meiotic metaphase I figures in two *Kosteletzkya* species and a tetraploid-tetraploid interspecific hybrid. **a** *Kosteletzkya adoensis* (76-36), 19_II_ **b** *Kosteletzkya borkouana* × *Kosteletzkya begoniifolia* (77-142), 22_II_ + 32_I_ **c** *Kosteletzkya borkouana* (76-40), 38_II_. Arrows in b. indicate two of the 22 univalents. Scale bar = 10 μm.

**Figure 3.**
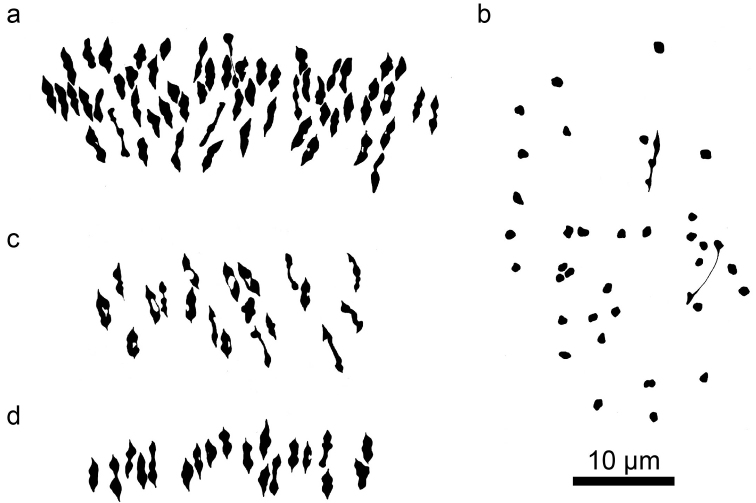
Camera lucida drawings of meiotic metaphase I figures in a species of *Kosteletzkya* and three diploid interspecific hybrids. **a** *Kosteletzkya racemosa* (82-88), 57_II_ **b** *Kosteletzkya depressa* × *Kosteletzkya adoensis* (77-136), 2_II_+34_I_ **c** *Kosteletzkya depressa* × *Kosteletzkya tubiflora* (75-149), 19_II_ **d** *Kosteletzkya buettneri* × *Kosteletzkya hispidula* (Sprengel, 1815) Garcke, 1881 (75-180), 19_II_.

The base chromosome number in *Kosteletzkya* is 19, and counts have been reported for 15 of the 17 species (Blanchard 1974, 2012; Table 1). The seven New-World species, with a center of diversity in Mexico, are all diploids. By contrast, the eight African species include three widely distributed diploids and, with more restricted distributions, four tetraploids (including the newly described *Kosteletzkya rotundalata* O. J. Blanchard, 2013 [Blanchard 2013]), and one hexaploid. Meiotic figures of representative diploid, tetraploid and hexaploid species are shown in Figs 2a, 2c and 3a.

**Table 1.** Chromosome numbers in *Kosteletzkya* (x = 19). Data from Blanchard 1974, 2012.

**New World Species**	**Chromosome number (n)**	**African Species**	**Chromosome number (n)**
*Kosteletzkya blanchardii* Fryxell, 1977	19	*Kosteletzkya adoensis* (A. Richard, 1847) Masters, 1868	19
*Kosteletzkya depressa* (Linnaeus, 1753) O. J. Blanchard, Fryxell et D. M. Bates, 1978	19	*Kosteletzkya buettneri* Gürke, 1889	19
*Kosteletzkya hispidula* (Sprengel, 1815) Garcke, 1881	19	*Kosteletzkya grantii* (Masters, 1868) Garcke, 1880	19
*Kosteletzkya pentacarpos* (Linnaeus, 1753) Ledebour, 1841	19	*Kosteletzkya begoniifolia* (Ulbrich, 1917) Ulbrich, 1924	38
*Kosteletzkya ramosa* Fryxell, 1977	19	*Kosteletzkya borkouana* Quézel, 1957	38
*Kosteletzkya reclinata* Fryxell, 1977	19	*Kosteletzkya rotundalata* O. J. Blanchard, 2013	38
*Kosteletzkya tubiflora* (de Candolle, 1824) O. J. Blanchard & McVaugh, 1978	19	*Kosteletzkya semota* O. J. Blanchard, 2008	37–38
		*Kosteletzkya racemosa* Hauman, 1961	57

The bi-centric geographical distribution of *Kosteletzkya* raises a question of where the group originated. Because the more complex, polyploid-rich species assemblage in Africa suggests a longer evolutionary history than its uniformly diploid New-World counterparts, I have speculated that Africa was the birthplace of the genus (Blanchard 2012).

Elsewhere in the Malvoideae, interspecific hybridization trials and the study of chromosome behavior in the resulting hybrids have been useful in clarifying species affinities, phytogeography and genomic differentiation. The two best-documented examples are the cotton genus *Gossypium* Linnaeus, 1753 and *Hibiscus* sect. *Furcaria* de Candolle, 1824.Each includes both diploid and polyploid species and each is distributed on both sides of the Atlantic, as well as in Australia. *Gossypium* comprises about 50 species (Fryxell in Verdcourt and Mwachala 2009) and eight distinct genomes (Fryxell 1992, Cronn and Wendel 2004), while *Hibiscus* sect. *Furcaria* boasts over 100 species and 13 identified genomes (Krapovickas 2006, Wilson 2006). With a few intriguing exceptions their genomes correspond to, or are confined to, distinct geographical areas.

Over a period of several years I have accumulated a living greenhouse collection of 15 *Kosteletzkya* species, and during that time I have incorporated them into a hybridization program that has attempted to shed light on the phytogeography and evolutionary history of the genus. The results are presented here. This information is expected in turn to illuminate molecular-level investigations of *Kosteletzkya* currently being pursued by the author and colleagues at the University of Florida.

## Materials and methods

Table 2 shows the greenhouse numbers, provenances and collectors of the 31 living accessions (i.e. cultivated progeny from a single seed source) of the 15 species used in this study, as well as six additional accessions from which the flowers in Fig. 6 were photographed.

**Table 2.** Sources of 38 greenhouse-grown accessions of *Kosteletzkya*. Thirty-two accessions, representing 15 species plus an artificial tetraploid, were used in the hybridization work. Six others were sources of some of the flowers photographed for Fig. 6. Species names are followed by one or more bolded greenhouse numbers in which the year of cultivation is indicated by the two digits preceding the hyphen.

***Kosteletzkya adoensis*,** **74-22,** ANGOLA: Huambo Distr., *Instituto de Investigação Agronómica de Angola s.n*.; **75-191,** SIERRA LEONE: Loma Mountains, on plateau at camp 2, *Morton SL 418*; ***7*6-36,** ETHIOPIA: Caffa a Bonga, *Saccardo 40*; **10-14**, ETHIOPIA: Gonder: Libo Awraja, ca. 15 km N of Addis Zemen,* Tadesse and Kagnew 1973*;**80-107,** MALAWI: N. Prov.: Nkhata Bay Dist.: 5 mi E of Mzuzu, *Pawek 11872*;**10-31,**MALAWI:Nkhata Bay Dist.: Vipya Plateau, 23 mi SW of Mzuzu, *Pawek 11275*;
***Kosteletzkya“art,”* 80-103, 82-90,** an artificial tetraploid, i.e. a spontaneously tetraploidized plant derived from an artificial hybrid between the two greenhouse plants *Kosteletzkya adoensis* 75-191 and *Kosteletzkya grantii* 76-2;
***Kosteletzkya begoniifolia*, 76-1, 10-64,** TANZANIA: Lerai Forest, Ngorongoro Crater, *Bonnefille and Riollet 73/26*;**79-21,** ETHIOPIA: ca. 40 km W of Ambo, *de Wilde and de Wilde-Duyfjes 10421*; **79-53,** KENYA: Seboti, SE Elgon, *Tweedie 3242*;
***Kosteletzkya blanchardii*,** **74-10, 76-21, 88-10, 90-19, 10-26,** MEXICO: Michoacán, 13 mi N of Tuzantla, *Fryxell, Bates and Blanchard 1650*;
***Kosteletzkya borkouana*, 76-40, 10-21,** UGANDA: “Rhino Camp,” Bahr el Jebel, Lado Enclave, *Mearns 2803*;**79-44,** CHAD: Borkou, Tigui, *Quézel s.n.*; **79-31, 90-36,** CONGO-KINSHASA: Plaine de la Ruzizi, Lac Tsimuka, *Germain 5682*;
***Kosteletzkya buettneri*, 74-11, 76-22, 88-6**, ZAMBIA: “C Province,” Kafue Pontoon, *Robinson 6706*; **90-27,** ZAMBIA: Chingola, *Handlos s.n*.; **90-25, 10-59,** TANZANIA: Buha Dist., Malagarasi Ferry, 40 mi. from Kibondo on Kasulu road, *Verdcourt 3444*;**79-28, 88-18,** CONGO-KINSHASA: Kipopo, près d’Elisabethville (Katanga), *Symoens 9242*; **90-8,** MALAWI: Bua River below Mude River confluence, *Robson 1542*;
***Kosteletzkya depressa*, 74-9, 75-136, 88-9, 90-17**, MEXICO: Nayarit: 30 mi. S of Compostela, *Fryxell, Bates and Blanchard 1563*;**10-18**, MEXICO:Sinaloa: between Rosario and Esquinapa,* Gentry, Barclay and Arguelles 19464*;
***Kosteletzkya grantii*,** **76-2,** CONGO-KINSHASA: Dungu, *Gérard 758*; **79-41, 89-71,** KENYA: Between Sio [“Soi”] River and Busia, *Evans and Erens 1655*;**10-104**, NIGERIA: Zaria: Jemaa, Sanga River Forest Reserve,* Keay 37217*;
***Kosteletzkya hispidula*, 74-6, 88-23, 90-39**, MEXICO: Sinaloa: S of Mazatlán, *Fryxell and Bates s.n*.; **10-44**, MEXICO: Sonora: N of El Sahuaral, *Felger and Reichenbacher 85-1581*;
***Kosteletzkya pentacarpos*, 74-19,** USA: Florida: Seminole Co., Lake Monroe N of Sanford, *Blanchard and Blanchard 306*; **88-8,** USA: Virginia: Chesterfield Co., N of Bermuda Hundred, *Harvill 17659*; **74-15,** USA: Florida: Sarasota Co., Laurel, *Blanchard and Blanchard 302*; **10-81,** IRAN: Astara [“Astava”], *Wright 62*; **80-142**, an intraspecific hybrid between the following two greenhouse plants**: **79-16,USA: Louisiana: Cameron Parish, Hackberry, *Blanchard and Blanchard 423*, and 79-38**,** IRAN: Astara [“Astava”], *Wright 62*;
***Kosteletzkya racemosa*, 79-24, 82-88, 90-5, 10-45,** CONGO-KINSHASA: Gandajika, *Liben 3266*;
***Kosteletzkya ramosa*, 88-1**, **10-23**, MEXICO: Jalisco: 1 mi E of Ayotlán, *Blanchard and Blanchard 1148*;
***Kosteletzkya reclinata*, 88-15, 10-5,** MEXICO: Jalisco: 11.7 km W of Tototlán, *Blanchard and Blanchard 1149*;
***Kosteletzkya rotundalata*, 80-104, 90-20,** **10-53**, CONGO-KINSHASA: Nizi, *Liben 444*;
***Kosteletzkya semota*, 90-2, 10-100,** NIGERIA: Ogun, Omi R., Ogun Makin, *Daramola s.n*.;
***Kosteletzkya tubiflora*,** **74-24, 90-11, 10-22,** MEXICO: Jalisco: NE of Guadalajara, Barranca de los Oblatos, *Fryxell, Bates and Blanchard 1590*; **76-23, 78-14**, MEXICO: Jalisco: K22 W of Guadalajara, *Fryxell and Bates 2137*.

Plants of *Kosteletzkya* grow readily under glass. Uniform germination was obtained by chipping away a bit of the seed coat at the radical end of the seed. When seeds were started in the spring, these mostly short-day plants came into flower in the following fall and winter.

### Cross-pollinations

In most species of *Kosteletzkya*, a flower persists for only a single day; by early afternoon it has already begun to wither. Cross-pollinations were therefore performed by hand in the morning, shortly after the flowers had fully opened. As has been reported for *Kosteletzkya pentacarpos* (Ruan et al. 2011), most species of *Kosteletzkya* recurve their styles later in the day and push their stigmas into the pollen mass, thereby effecting self-pollination in the absence of any earlier exogenous pollination. This creates a problem when controlled crosses are attempted because it is impossible to be certain that successful seed-set was due to pollen from the other experimental parent rather than from the same flower. In the case of the larger, sturdier flowers found in some other Malvoideae, the pre-anthesis removal of the anthers solves this problem. This has been done, for example, in *Hibiscus* sect. *Furcaria* (Menzel and Wilson 1961), *Hibiscus* sect. *Muenchhusia* (Fabricius, 1763) O. J. Blanchard, 1988 (Wise and Menzel 1971), and *Gossypium* (Wilson and Stapp 1985). However the same technique was found to be too traumatic and inefficient for smaller flowers such as those of *Kosteletzkya*.

A solution to this problem was found in the use of halves of gelatin capsules to separate the male and female parts of the flowers (Fig. 1a). In this technique a hole is punctured through the apex of a capsule-half using a dissecting needle that has been heated in a flame. A razor blade is then used to cut up one side of the capsule-half wall and over the top, passing through the previously cut hole. It is then possible to 1) insert the closed tines of a straight forceps into the open end of the capsule-half, 2) allow the tines to spread the razor-cut slit, 3) slip the whole unit over the base of the style branches distal to the anther mass, and then 4) allow the capsule-half to close, effectively isolating the stigmas and pollen mass from one another (Fig. 1b). In the present study each such unit was attached to a flower immediately after a cross-pollination, and the procedure proved to be highly successful in preventing self-pollination. The only drawback was that extra care had to be taken when watering the plants in order to avoid deforming or dissolving the capsule-halves.

The gelatin-capsule device could not be used with one of the species. *Kosteletzkya borkouana* Quézel, 1957 is effectively an obligate selfer because the stigmas have usually already recurved into the pollen mass by the time the corolla opens in the morning. To make matters more difficult, this species has the smallest flowers of any *Kosteletzkya*. Nevertheless it was necessary to visit the plants at 0300-0400h to carefully cut away the unopened corolla and remove the mercifully few pre-dehiscence anthers. And because the time was still hours away from the anthesis of any of the other species, actual manual cross-pollination of the emasculated flower had to await a later visit.

While most of the species that were studied were short-day, fall-and-winter-flowering plants, three of them flowered in the late summer (*Kosteletzkya pentacarpos)* or early fall (*Kosteletzkya ramosa* Fryxell, 1977 and *Kosteletzkya reclinata* Fryxell, 1977). To make these three available to a greater variety of other potential crossing partners, beginning in early summer plants of several of the other species were put on carts, moved daily at 1700h into an adjoining darkened room, and retrieved the next morning. Within three or four weeks, flower-bud initiation was evident. Early flowering was thereby induced so as to coincide with the flowering of the three late-summer-early-fall species.

In general, crosses worked in both directions. It appeared to make no difference in the success of an attempted cross whether a participant in the cross was the ovule-parent or pollen-parent, so no tabulated distinction is made here concerning the direction of the crosses reported. As a matter of insurance, however, the actual practice was that whenever two plants were crossed in which the size of the flowers, or more especially the style lengths, were considerably different, the smaller of the pair was used as the pollen recipient, on the theory that pollen adapted to traversing a short style might be challenged by a longer style (Williams and Rouse 1990, Sorensson and Brewbaker 1994, Tiffin et al. 2001).

Voucher specimens of most of the plants used as parents of crosses in this study, as well as specimens of the hybrids themselves, are deposited at the University of Florida Herbarium (FLAS), Florida Museum of Natural History, Gainesville, Florida, USA. In a few cases the vouchers for the parents are either the original wild-collected specimens that were the seed sources for the greenhouse plants, or they are specimens from the same seed source but grown in other years.

### Pollen stainability

Pollen was stained with Cotton Blue in lactophenol. Each pollen slide was made from a single flower. Normal-sized, fully and deeply blue-stained grains were treated as “stained” and are expressed here as a percent of the total number of grains on a slide. At least three and usually five or more slides were counted for each hybrid combination. The majority of the species that participated as parents in the stainability evaluations were themselves counted and their stainabilities were found to range from 97 to 100 percent. Later in the hybridization program space was at a premium and the few replicates of hybrid plants that could be grown were used almost solely as a source of young flower buds for meiotic samples, so for some of the later-produced hybrids no fruit or pollen data were obtained. By that time, however, the general patterns of fruit-set and pollen stainability were already evident.

### Fruit-set

Recurvature of the styles is a problem for controlled pollinations, but it is a boon for fruit-set purposes because, with one exception, it was theoretically possible to let the greenhouse hybrids pollinate themselves and use those results rather than resorting to manual self-pollination. In actual practice, however, the plants were usually hand-pollinated anyway, as a part of the routine of nearly daily visits to the greenhouse.

The exception is hybrid progeny in which *Kosteletzkya tubiflora* (de Candolle, 1824) O. J. Blanchard & McVaugh, 1978 is one parent. This species bears distinctive yellow-and-red tubular flowers with an exserted staminal column and style (Figure 6G), and they are almost certainly bird-pollinated in the plant’s native setting. The related *Kosteletzkya thurberi* A. Gray, 1887 with structurally similar flowers, was reported to be visited by the Bumblebee Hummingbird (*Atthis heloisa* [Lesson and DeLattre, 1839]; reported as “*Selasphorus heloisa*”) in northern Mexico (Van Devender et al. 2004). Unlike the rest of the species, *Kosteletzkya tubiflora* has protogynous flowers that remain open and nectar-producing for two days or more. On the first day the stigmas are receptive and the anthers remain undehisced. By the next day the staminal column has elongated, exserting the now-dehiscent anthers to the earlier position of the stigmas. At this point, the stigmas may or may not remain receptive, but they do not recurve in the absence of pollination. As a consequence, the hybrids involving this species required hand-pollination.

Fruits will set in *Kosteletzkya* when as few as one of the five ovules has been fertilized, and no distinction is made here as to the number of seeds in a set fruit. When an unfertilized spent flower falls, part of the pedicel remains attached to the plant, readily marking the former presence of a flower. Percent fruit-set is simply the proportion of fruit-bearing pedicels out of a total number of post-flowering pedicels.

### Chromosome pairing

**Figure 4.**
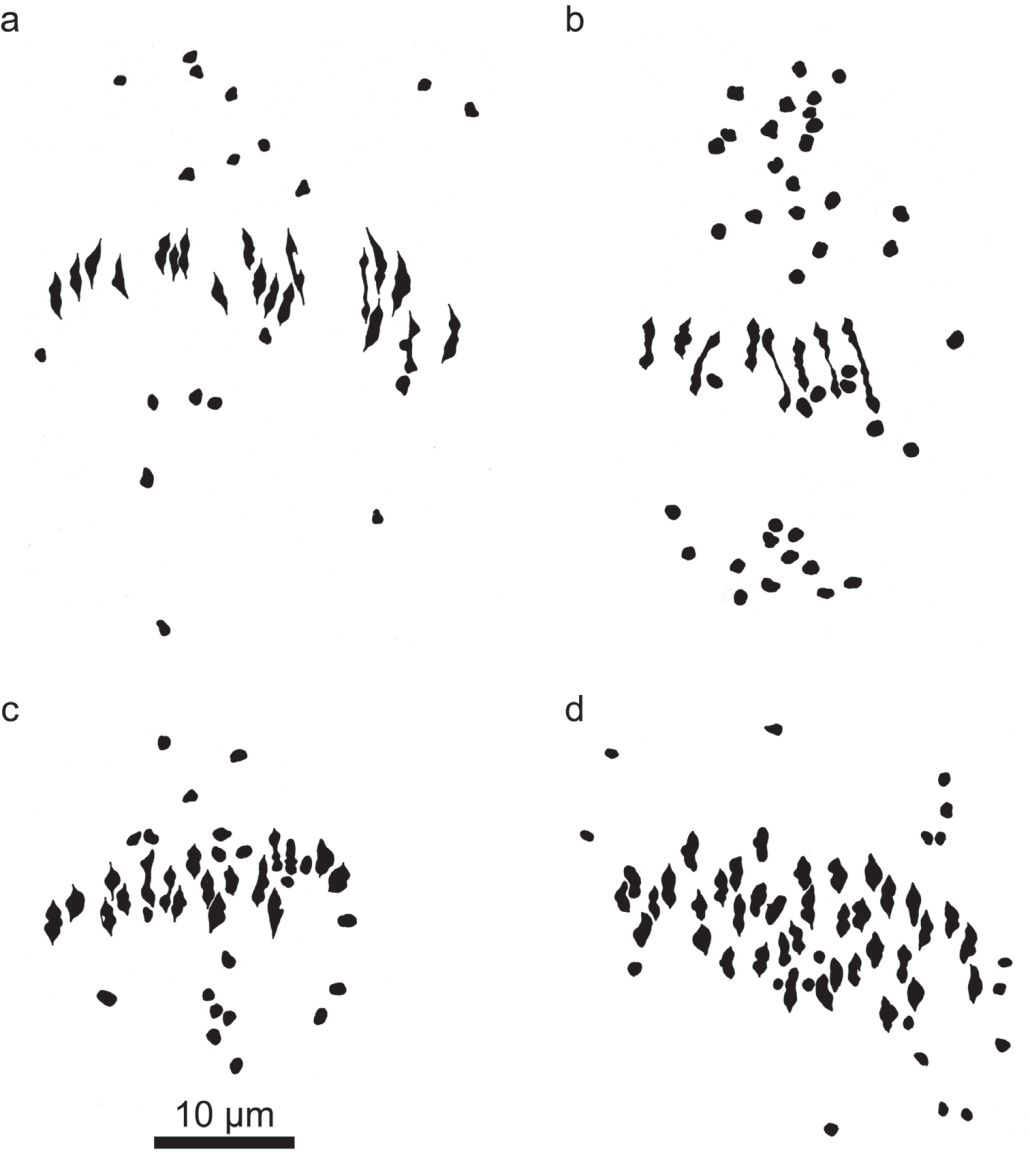
Camera lucida drawings of meiotic metaphase I figures in *Kosteletzkya* interspecific hybrids in which at least one parent is a polyploid. **a** *Kosteletzkya buettneri* × *Kosteletzkya borkouana* (77-160), 19_II_+19_I_ **b** *Kosteletzkya grantii* × *Kosteletzkya borkouana* (77-166), 8_II_+41_I_ **c** *Kosteletzkya grantii* × *Kosteletzkya begoniifolia* (81-70), 18_II_+21_I_ **d** *Kosteletzkya begoniifolia* × *Kosteletzkya racemosa* (80-119), 38_II_+19_I_.

**Figure 5.**
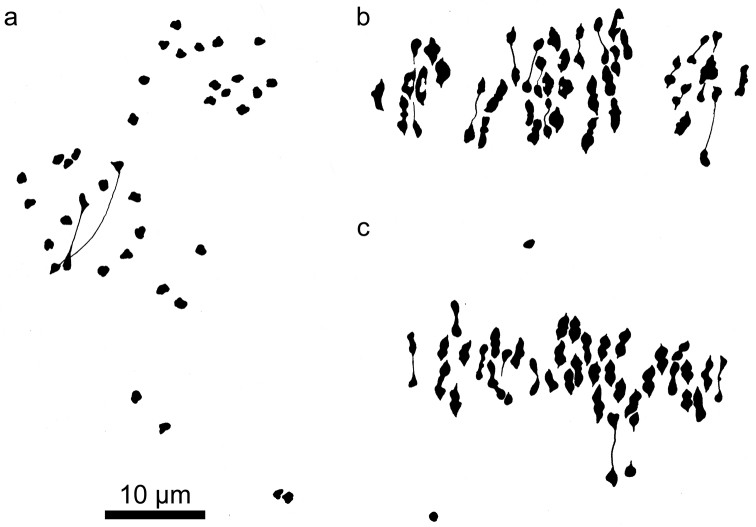
Camera lucida drawings of meiotic metaphase I figures in *Kosteletzkya* interspecific hybrids and an artificial tetraploid. **a** *Kosteletzkya adoensis* × *Kosteletzkya grantii* (77-153), 2_II_+34_I_ **b** artificial tetraploid (82-90), 38_II_ **c** *Kosteletzkya* artificial tetraploid × *Kosteletzkya begoniifolia* (81-79), 37_II_+2_I_.

All chromosome counts and observations of chromosome pairing were made from pollen mother cells (PMCs) at meiotic metaphase I. Details of methods of collection, fixing, staining, and preservation of meiotic material can be found in Bates and Blanchard (1970) and Blanchard (2012). It was seldom possible to obtain preparations in which all of the chromosomes were in the same plane of focus. This was not a problem for microscopic examination and interpretation, but it usually yielded less-than-satisfactory photographs (Figure 2a–c), especially of hybrids with numerous univalents that were not constrained at the metaphase plate (Fig. 2b); hence the extensive use here of camera lucida drawings. For simplicity of presentation, and because of the scale of this study, cytological outcomes are expressed in bivalent-equivalents in which the occasional quadrivalent is converted to two bivalent-equivalents, and the occasional trivalent is expressed as a single bivalent-equivalent. Unpaired chromosomes (univalents) were encountered in all crosses between different ploidy levels (e.g. Fig. 4a–d), as well as when the parents of a cross are genetically substantially divergent (e.g. Fig. 2b, 3b, 5a), and these too were counted, at least earlier in the investigation. Again, however, for the sake of clarity, they are not tabulated, but should be understood to have been present. For most hybrids at least five PMCs were examined; in more that half of the cases, more than 10 were examined.

## Results

A total of 56 interspecific hybrid combinations were obtained during the course of the study (Table 3). Mean values and numbers of observations are presented for percent pollen stainability, percent fruit-set and number of chromosome bivalent-equivalents in these hybrids.

**Table 3.** Experimental crosses among species of *Kosteletzkya*, showing meanvalues and numbers of observations (N) for pollen stainability, fruit-set and chromosome pairing in the hybrids. Hybrids are divided into New-World, African, and trans-Atlantic crosses, plus crosses with an artificial tetraploid. Species names are abbreviated using the first three letters of the specific epithet of species listed in Table 1. Names of polyploid taxa are bolded. The maximum potential number of chromosome pairs for any particular hybrid combination is also shown, in bold, in the right-most column of the table. Note that in a few cases a particular interspecific hybrid combination was made using more than one specific pair of parent plants (see, for example, bue × **sem**).

**cross**	**parental greenhouse numbers**	**hybrid greenhouse number**	**% pollen stainability**		**% fruit set**		**bivalent equivalents**		**maximum potential pairing**
			**mean**	**N**	**mean**	**N**	**mean**	**N**	
**NEW WORLD**									
bla × dep	74-9 × 74-10	75-144	**42**	7	**20**	584	**19**	16	**19**
bla × his	74-6 × 74-10	75-135	**86**	5	**27**	724	**19**	13	**19**
bla × pen	74-19 × 74-10	75-159	**15**	6	**2**	127	**18.9**	10	**19**
bla × ram	88-10 × 88-1	89-5	**90**	3	**7**	71	**19**	6	**19**
bla × tub	74-24 × 4-10	75-161	**56**	6	**25**	4	**18.7**	6	**19**
dep × pen	74-19 × 4-9	75-150	**25**	5	**14**	224	**18.5**	13	**19**
dep × his	74-9 × 74-6	75-178	**39**	5	**41**	758	**18.9**	11	**19**
dep × ram	88-9 × 88-1	89-11	**39**	3	**26**	186	**-**	-	
dep × rec	88-9 × 88-15	89-12	**51**	3	**49**	390	**19**	3	**19**
dep × tub	74-9 × 74-24	75-149	**51**	5	**29**	143	**19**	25	**19**
his × pen	74-6 × 74-15	75-127, 75-157	**79**	5	**11**	210	**18.9**	14	**19**
his × ram	88-23 × 88-1	89-7	**95**	3	**65**	147	**-**	-	
his × rec	88-23 × 88-15	89-1	**94**	3	**54**	247	**19**	8	**19**
his × tub	74-6 × 74-24	75-151	**91**	6	**49**	138	**19**	17	**19**
pen × rec	88-8 × 88-15	89-6	**78**	3	**38**	343	**18.8**	9	**19**
pen × tub	74-19 × 74-24	75-168	**73**	6	**31**	88	**18.9**	9	**19**
rec × ram	88-15 × 88-1	89-3, 89-9	**98**	6	**66**	154	**18.9**	7	**19**
**AFRICA** (polyploid species **bolded**)									
ado × bue	76-36 × 76-22	77-145	**0**	5	**0**	113	**3.1**	10	**19**
ado × gra	75-191 × 76-2	77-153	**1**	5	**0**	37	**2.0**	25	**19**
bue × gra	76-2 × 76-22	77-158	**0**	5	**0**	112	**9.1**	17	**19**
bue × **beg**	76-22 × 76-1	77-100, 81-26	**0**	5	**0**	252	**3.9**	25	**19**
	79-28 × 79-53	81-31	**-**	-	**0**	218	**-**	-	
bue × **bor**	76-22 × 76-40	77-160	**0**	5	**0**	21	**18.8**	12	**19**
bue × **sem**	90-27 × 90-2	91-34	**-**	-	**-**	-	**8.5**	6	**19**
	90-8 × 90-2	91-39	**-**	-	**-**	-	**4.4**	25	**19**
	90-25 × 90-2	91-10	**-**	-	**-**	-	**6.1**	15	**19**
gra × **beg**	79-41 × 79-21	81-70, 81-74	**2**	3	**0**	247	**17.9**	15	**19**
gra × **bor**	76-2 × 76-40	77-166	**0**	5	**0**	10	**7.0**	3	**19**
gra × **sem**	90-2 × 89-71	91-37	**-**	-	**-**	-	**13.1**	9	**19**
**beg** × **bor**	76-1 × 76-40	77-142	**5**	5	**0**	171	**24.6**	14	**38**
**beg** × **rot**	79-53 × 80-104	81-76	**97**	4	**63**	182	**37.0**	2	**38**
**bor × sem**	90-36 × 90-2	91-8	**-**	-	**-**	-	**3.5**	15	**37-38**
**rot × sem**	90-2 × 90-20	91-4	**-**	-	**-**	-	**11.3**	20	**37-38**
**beg × rac**	79-21 × 79-24	80-119	**42**	5	**0**	234	**37.1**	9	**38**
**bor × rac**	79-44 × 79-24	80-115, 80-116	**5**	5	**0**	671	**37.8**	4	**38**
	79-31 × 79-24	80-117	**-**	-	**0**	147	**-**	-	
**rot × rac**	80-104 × 79-24	81-73	**33**	3	**0**	141	**-**	-	
**sem × rac**	90-2 × 90-5	91-3	**-**	-	**-**	-	**6.6**	11	**37-38**
**TRANS-ATLANTIC** (African species listed first; polyploid species **bolded**)									
ado × dep	74-22 × 74-9	75-130, 75-156, 77-136	**7**	8	**1**	143	**1.2**	13	**19**
bue × bla	74-11 × 74-10	75-145	**26**	10	**1**	155	**19**	10	**19**
bue × dep	74-11 × 74-9	75-104	**32**	14	**2**	264	**18.9**	13	**19**
bue × his	74-11 × 74-6	75-180	**37**	5	**8**	245	**19**	10	**19**
bue × pen	74-11 × 74-19	75-148	**27**	7	**0**	129	**19**	11	**19**
bue × ram	88-18 × 88-1	89-8	**74**	3	**14**	29	**18.9**	8	**19**
bue × rec	88-6 × 88-15	89-4	**35**	3	**0**	47	**-**	-	
bue × tub	79-28 × 78-14	81-91	**61**	3	**4**	75	**19**	5	**19**
gra × bla	76-2 × 76-21	77-113	**0**	5	**0**	791	**11.4**	10	**19**
gra × dep	76-2 × 75-136	77-115	**-**	-	**-**	-	**7.7**	13	**19**
gra × pen	79-41 × 80-142	81-159	**0**	3	**0**	75	**-**	-	
**beg** × bla	76-1 × 76-21	77-104	**1**	4	**0**	272	**6.1**	14	**19**
**bor** × dep	76-40 × 75-136	77-183	**4**	5	**0**	67	**18.0**	1	**19**
**bor** × bla	76-40 × 76-21	77-167	**-**	-	**-**	-	**18.6**	11	**19**
**bor** × tub	76-40 × 76-23	77-173	**0**	5	**0**	17	**18.7**	6	**19**
**sem** × bla	90-2 × 90-19	91-1	**-**	-	**-**	-	**4.3**	19	**19**
**sem** × his	90-2 × 90-39	91-35	**-**	-	**-**	-	**7.0**	17	**19**
**sem** × tub	90-2 × 90-11	91-36	**-**	-	**-**	-	**10.9**	15	**19**
**CROSSES WITH ARTIFICIAL TETRAPLOID** (polyploid taxa **bolded**)									
**art** × ado	80-103 × 80-107	81-89	-		**-**		**19**	4	**19**
**art** × gra	80-103 × 79-41	81-87	-		**-**		**19**	5	**19**
**art × beg**	80-103 × 79-53	81-79	-		**-**		**36.9**	13	**38**
**art × rot**	80-103 × 80-104	81-83	-		**-**		**36.6**	15	**38**

### New-World interspecific crosses

All seven available New World species were involved in the crossing program (an eighth species, *Kosteletzkya thurberi*, was unavailable), and all interspecific crosses that were attempted were successful and comprised 17 of the 21 possible pairwise combinations among the seven species. Hybrid plants generally grew as vigorously as their parents under greenhouse conditions. Pollen stainability among them ranged from 15 to 98 percent, while fruit-set ranged from 2 to 66 percent. Low or high values in one measure did not necessarily correspond with those of the other measure. For instance, the combination *Kosteletzkya blanchardii* Fryxell, 1977 × *Kosteletzkya ramosa* had 90 percent pollen stainability but only 7 percent fruit-set.

**Figure 6.**
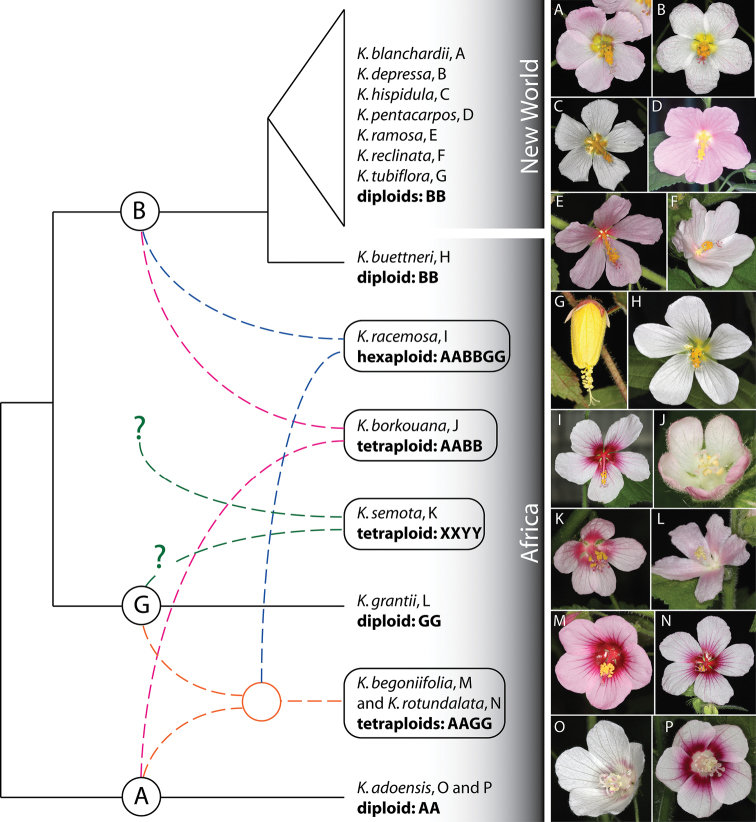
A representation of hypothesized phylogenetic and phytogeographic relationships among 15 species of *Kosteletzkya* based on chromosome pairing in experimental hybrids, plus photographs of the 15 species. Extant allopolyploids are shown in boxes. Distinct genomes are identified by the letters A, B, G, X and Y. Differently colored dashed lines indicate how these ancestral parents are thought to have combined to form the present-day polyploid species. One of the two alternate derivations of *Kosteletzkya racemosa*—that in which the B genome is carried by the diploid ancestor—is shown here. Question marks indicate uncertainties about the origins of the postulated diploid ancestors of *Kosteletzkya semota*. Letters after the names of taxa refer to the flower photographs to the right. The latter are not all shown to the same scale. Plants from which the flower images were made are indicated by the following greenhouse numbers (see Table 2): **A** 10-26 **B** 10-18 **C** 10-44 **D** 10-81 **E** 10-23 **F** 10-5 **G** 10-22 **H** 10-59 **I** 10-45 **J** 10-21 **K **10-100 **L** 10-104 **M** 10-64 **N** 10-53 **O** 10-14 **P** 10-31.

Despite wide morphological differences among the New-World species, nearly complete chromosome pairing, as indicated by the average number of bivalents, was found in each of the 15 hybrid combinations that were examined meiotically (Table 3). Average values ranged from 18.5 to 19 bivalents out of a possible 19. As an example, Fig. 3c shows a meiotic metaphase figure from the hybrid *Kosteletzkya depressa* (Linnaeus, 1753) O. J. Blanchard, Fryxell et D. M. Bates, 1978 × *Kosteletzkya tubiflora*, whose parental species are dramatically different in habitat, morphology and floral adaptations. *Kosteletzkya depressa* is a lowland, bee-pollinated plant with a small, white-to-pink, rotate corolla (petals 0.8–1 cm long), included staminal column, and a green calyx (Fig. 6B); *Kosteletzkya tubiflora* is an upland, apparently bird-pollinated plant with a large, yellow, tubular corolla (petals 2.5–3 cm long), exserted staminal column and a pink-to-red calyx (Fig. 6G).The two species also differ markedly in fruit and seed characteristics.

### African interspecific crosses

All three possible hybrids among the three known African diploid species *Kosteletzkya adoensis* (A. Richard, 1847) Masters, 1868, *Kosteletzkya buettneri* and *Kosteletzkya grantii* (Masters, 1868) Garcke, 1880 were obtained, although these offspring were not as robust as the New-World hybrids. Like the New-World diploid species, the three African parent species differ considerably in habit, leaf shape, inflorescence form and details of flowers (see Figs 6H, L, O and P), fruits and seeds. However in dramatic contrast to the diploid New-World hybrids, pollen stainability in the African diploid hybrids ranged from 0 to 1 percent, fruit-set was 0 percent, and average chromosome pairing ranged from 2.0 to 9.1 out of a potential 19 bivalent-equivalents. A meiotic metaphase I of the hybrid *Kosteletzkya adoensis* × *Kosteletzkya grantii* is shown in Fig. 5a.

Hybrids between African diploids and African tetraploids were generally obtained with more difficulty, particularly in the case of the diploid *Kosteletzkya adoensis*, in which, to cite the most extreme example, several hundred cross-pollinations with *Kosteletzkya begoniifolia* (Ulbrich, 1917) Ulbrich, 1924 resulted in only a single viable seed. Nevertheless, altogether six of the possible 12 diploid-tetraploid hybrids were eventually produced. As might be expected in hybrids between ploidy levels, pollen stainability was low (0-2 percent in the four interspecific combinations that were sampled for this characteristic) and their fruit-set was likewise low (0 percent in the same four combinations). Average chromosome pairing in these six hybrids varied widely. Two approached the potential maximum of 19, forming 17.9 to 18.8 pairs (Figs 4a and 4c), while the other four ranged in average from 3.9 to 13.1 bivalent-equivalents (Fig. 4b).

No diploid-hexaploid hybrids could be obtained despite numerous cross-pollinations.

The African polyploids could be crossed fairly easily among themselves, and eight of the ten possible hybrids were produced. In the five cases where pollen stainability and fruit-set were determined, stainability ranged from 5 to 97 percent, whereas fruit-set for four of the hybrids was 0 percent, while for a fifth (*Kosteletzkya begoniifolia* × *Kosteletzkya rotundalata*) it was 63 percent. Of the four tetraploid-tetraploid hybrids that were examined cytologically, one had bivalent-equivalents approaching the maximum possible 38, whereas the other three ranged from 3.5 to 24.6 bivalent-equivalents (Fig. 2b). Of the three tetraploid-hexaploid hybrids examined cytologically, two averaged over 37 pairs out of a potential maximum of 38 (Fig. 4d), while the third averaged only 6.5 bivalent-equivalents.

### Trans-Atlantic interspecific crosses

Eleven of the 21 possible diploid-diploid trans-Atlantic combinations were produced. At least four failed attempts involved *Kosteletzkya adoensis* as one potential parent. One combination that was obtained, *Kosteletzkyagrantii* × *Kosteletzkya depressa*, produced flower buds that aborted between meiosis and flowering, making pollen stainability and fruit-set data impossible to obtain. In another combination, *Kosteletzkya adoensis* × *Kosteletzkya depressa*, though the plants flowered, they were weak-stemmed and slow-growing, and produced only a feeble root system. The 10 surviving hybrids had pollen stainabilities and fruit-sets that were intermediate, on average, between those of New World diploid-diploid crosses and those of the African diploid-diploid crosses, and ranged from 0 to 74 percent pollen stainability and 0 to 14 percent fruit-set. However, depending on which of the African diploids participated, the pollen-stainability outcomes were different: in the seven African-New World crosses involving *Kosteletzkya buettneri*, stainability ranged from 26 to 74 percent; in the two crosses involving *Kosteletzkya grantii*, stainability was zero percent in both cases; and in the single cross involving *Kosteletzkya adoensis*, the result was seven percent pollen stainability. Outcomes of chromosome pairing observations were even more distinctly different depending on which African parent was involved. In all six combinations in which *Kosteletzkya buettneri* was the African parent, pairing closely approached the maximum possible 19 (see for example Fig. 3d). However when *Kosteletzkya grantii* or *Kosteletzkya adoensis* were involved, the pairing in the three hybrids that were examined cytologically ranged from 1.2 to 11.4 bivalent-equivalents (see for example Fig. 3b).

In the case of trans-Atlantic crosses between African tetraploids and New-World diploids there was again a bimodal pattern. In three of the seven hybrid combinations examined meiotically, chromosome pairing approached the maximum 19; in the other four the range was 4.3 to 10.9.

As was the case for African-African crosses, no diploid-hexaploid trans-Atlantic combinations could be obtained despite numerous crossing attempts.

## Discussion

### Genome differentiation and identification

In clear contrast to the nearly perfect chromosome pairing (18.5–19 bivalent-equivalents) in all of the 17 diploid New-World hybrids, the three African diploids contain chromosome sets with only low-to-modest affinity among themselves (2.0, 3.1 and 9.1 bivalent-equivalents). A consequence of this is mirrored in the negligible pollen stainability (0 to 1 percent) and zero fruit-set in hybrids among the three African species. This has prompted the designation here of three distinct genomes among the African diploids: A for the *Kosteletzkya adoensis* genome, B for the *Kosteletzkya buettneri* genome and G for the *Kosteletzkya grantii* genome.

Considering chromosome-pairing relationships in these genomic terms, it is apparent that only one genome is shared by all of the New World species. More interestingly, the trans-Atlantic crosses between the African diploid *Kosteletzkya buettneri* and six different species from the New World show a nearly perfect pairing in each, consisting on average of 18.9 to 19 bivalent-equivalents. Clearly then, the one New-World genome must be B. Indirect support for this comes from the fact that New-World species recognize only 7.7 to 11.4chromosomes in the G genome, and only 1.2 chromosomes in the A genome—a pattern similar to crosses of these same two genomes directly with *Kosteletzkya buettneri* itself. These results, of course, indicate a direct connection between the African and New World parts of the genus. Simply stated, the African *Kosteletzkya buettneri* appears to be more closely related to the New World species than it is to its two African diploid congeners.

### Genomes and polyploids

Allopolyploidy is an important mechanism for speciation in plants (Otto and Whitton 2000, Soltis et al. 2004, Wood et al. 2009), and polyploid series among related species are often found to have resulted from this process. In the Malvaceae: Malvoideae, allopolyploidy has been extensively documented in *Gossypium* (Endrizzi et al. 1985) and in *Hibiscus* sect. *Furcaria* (Wilson 1994). In *Hibiscus* sect. *Furcaria*, all of the 41 genomically studied polyploid species are allopolyploids; in *Gossypium*, all of the five known polyploid species are allopolyploids. On this basis I have hypothesized that the polyploid species in *Kosteletzkya* will prove to be allopolyploids as well.

In classic allopolyploidy, the production of an interspecific hybrid is the first step leading to a new species, yet in an examination of over 2800 herbarium specimens comprising all 17 species in the genus, no plants were found that might have been considered natural hybrids. In a way, this is not a surprise. The New-World species, though relatively easily inter-crossable, are at present largely geographically allopatric, and even where they are in geographic proximity they are kept separate elevationally (e.g. *Kosteletzkya depressa* and *Kosteletzkya tubiflora* in western Mexico) or by flowering season (e.g. *Kosteletzkya depressa* and *Kosteletzkya pentacarpos* in western Cuba and southern Florida). In contrast, while the African diploids are broadly sympatric, though perhaps often ecologically separated, experiments reported here have shown that hybrids among them are more difficult to obtain and weaker—conditions likely to pertain in the wild as well. In either hemisphere the hybrids themselves would obviously be transitory, living for a few years and then likely vanishing unrecognized and leaving few if any progeny.

Nevertheless, there appears to be clear evidence of allopolyploidy in Africa. The experimental diploid-tetraploid hybrid between *Kosteletzkya grantii* (2x) and *Kosteletzkya begoniifolia* (4x) averaged 17.9 bivalent-equivalents out of a possible 19, and likewise the diploid-tetraploid hybrid between *Kosteletzkya buettneri* (2x) and *Kosteletzkya borkouana* (4x) averaged 18.8 out of 19. The reverse crosses (*Kosteletzkya buettneri* × *Kosteletzkya begoniifolia* and *Kosteletzkya grantii* × *Kosteletzkya borkouana*) yielded averages of 3.9 and 7.0 respectively. This suggests that the G genome but not the B genome is present in the tetraploid *Kosteletzkya begoniifolia*, and the B genome but not the G genome is present in the tetraploid *Kosteletzkya borkouana*. Neither of these two diploids was combined in this study into a hybrid with the tetraploid *Kosteletzkya rotundalata* but this species shows nearly complete chromosome homology with *Kosteletzkya begoniifolia*—an average of 37.0 pairs out of a potential 38—suggesting a close relationship between the two, and indirectly indicating that the G genome is also present in *Kosteletzkya rotundalata*. Finally, the tetraploid *Kosteletzkya semota* was combined with both the B-bearing *Kosteletzkya borkouana* and the G-bearing *Kosteletzkya rotundalata*, producing on average only 3.5 and 11.3 pairs respectively, from which it can be reasonably concluded that *Kosteletzkya semota* contains neither B nor G genomes. This is further suggested by crosses of *Kosteletzkya semota* with the African diploids *Kosteletzkya buettneri* and *Kosteletzkya grantii* as well as with three New-World B-genome diploids, in which all five results ranged between 4.3 and 13.1 bivalent-equivalents.

When the tetraploids *Kosteletzkya begoniifolia* and *Kosteletzkya borkouana* were crossed with one another, the resulting hybrids averaged 24.6 bivalent-equivalents out of a possible 36, which indicates that they share a genome. That genome cannot be either G or B since it is shown above that neither is shared by the two tetraploids. This shared, unknown *Kosteletzkya borkouana*-*Kosteletzkya begoniifolia*-*Kosteletzkya rotundalata* genome cannot be present in *Kosteletzkya semota* because many fewer that a full set of 19 chromosomes were detected in *Kosteletzkya semota* by these other tetraploids.

### The artificial tetraploid

Although one might invoke some undiscovered or now-extinct genome as the postulated shared genome, the most obvious suggestion is that it is the extant genome A. It was therefore particularly frustrating that hundreds of cross-pollinations between the A-bearing *Kosteletzkya adoensis* and the tetraploids *Kosteletzkya begoniifolia* and *Kosteletzkya borkouana* produced only a single viable seed—from the first of these two combinations—and that seed yielded a severely stunted, deformed, non-reproductive plant. This made it impossible to introduce an A genome into either of the two tetraploids to the extent that it could reach meiosis and seek out a possible genomic match. On the other hand, the putative hybrids that would have had to form in nature as a first step in the allopolyploid production of the postulated AAGG and AABB tetraploids are, respectively, the combinations AG (*Kosteletzkya adoensis* × *Kosteletzkya grantii*), and AB (*Kosteletzkya adoensis* × *Kosteletzkya buettneri*). Both of these hybrids were indeed produced experimentally. The first had 1 percent pollen stainability, 0 percent fruit-set and produced an average of 2.0 bivalent-equivalents out of a potential 19; the second had 0 percent pollen stainability, 0 percent fruit-set, and an average of 3.1 bivalent equivalents. Fig. 5a illustrates a meiotic metaphase I of the AG hybrid.

Remarkably, one day I found in the greenhouse a normal-appearing fruit on a branch of the otherwise profoundly sterile diploid hybrid AG plant. It contained three well-formed seeds, all of which subsequently germinated and produced vigorous plants which themselves yielded abundant fruit when selfed. This restoration of fertility strongly suggested that a spontaneous doubling of chromosomes had occurred in at least a small part of the hybrid plant, thereby creating identical pairs of chromosome sets and permitting full synapsis in meiotic prophase I, with the result that meiosis and gamete formation were able to proceed to normal completion. Examination of meiotic metaphase in one of these plants indeed showed 38 pairs of chromosomes (Fig. 5b). The conclusion: a combination of experimental and accidental events had resulted in a new, fully fertile tetraploid. A test of this would be a cross between this artificial tetraploid and one of its wild putative counterparts, *Kosteletzkya begoniifolia* or *Kosteletzkya rotundalata*. Both of these test hybrids were obtained, and they showed averages of 36.9 and 36.6 bivalent-equivalents respectively out of a potential 38. Fig. 5c shows a meiotic figure illustrating the combination *Kosteletzkya begoniifolia* × *Kosteletzkya* artificial tetraploid.

This settled two important matters: 1) that the artificial tetraploid corresponded genomically to these wild tetraploids, and 2) that the identity of the elusive shared genome was indeed A. The latter was also separately verified by subsequent backcrossing of the artificial tetraploid to each of its two diploid parents. These crosses with *Kosteletzkya adoensis* and with *Kosteletzkya grantii* each yielded an average pairing of 19 out of 19 potential pairs. Interestingly, *Kosteletzkya adoensis*, which had been so intractable in attempts at crossing it with the two wild tetraploids, crossed fairly readily with their home-made counterpart, and the offspring grew and flowered well.

In summary, these results suggest that the genomic makeups of the tetraploids *Kosteletzkya begoniifolia*, *Kosteletzkya rotundalata* and *Kosteletzkya borkouana* are respectively AAGG, AAGG and AABB.

Since it is generally observed that most allopolyploids arise via unreduced gametes (de Wet 1971, Ramsey and Schemske 1998), and since the actual initiation of an allopolyploid event is rarely witnessed, it is noteworthy that the spontaneous polyploidization reported here was apparently due not to unreduced gametes but to somatic doubling. Part of the plant—perhaps only a single flower—must have arisen from a chromosome doubling in a somatic apical initial or an early derivative. A contrary interpretation would require the unlikely independent production, within only a single flower and no other flowers on the plant, of a minimum of six unreduced gametes—three eggs and three sperm—which then would have to meet by chance and go on to result in a single capsule bearing three fertile seeds out of a potential five.

### The hexaploid Kosteletzkya racemosa Hauman, 1961

Despite numerous attempts, no hybrids could be produced between the single African hexaploid *Kosteletzkya racemosa* and any of the diploids, either African or New-World, however the hexaploid did cross with all four tetraploids. With *Kosteletzkya begoniifolia* and *Kosteletzkya borkouana* it averaged 37.1 and 37.8 chromosome pairs respectively out of a possible 38 (Figure 4d), whereas with *Kosteletzkya semota* it showed 6.6 pairs out of a possible 38. (Meiotic material from the hybrid *Kosteletzkya rotundalata* × *Kosteletzkya racemosa* was not obtained.) Now that genomic makeups are known for the tetraploids *Kosteletzkya borkouana* and *Kosteletzkya begoniifolia* it can be stated with reasonable certainty that the genomic constitution of the hexaploid is AABBGG. This is because both AAGG (*Kosteletzkya begoniifolia*) and AABB (*Kosteletzkya borkouana*) tetraploids were shown separately to find nearly perfect correspondence with two sets of chromosomes in the hexaploid.

The hexaploid could theoretically have arisen in nature in one of two ways. A triploid ABG hybrid could have formed between an AABB tetraploid and a GG diploid, or between an AAGG tetraploid and a BB diploid, in both cases followed by chromosome doubling. No persuasive evidence at present strongly favors either scenario as being more likely, but the *Kosteletzkya buettneri*-like narrower leaves and depressed fruit, plus the *Kosteletzkya begoniifolia*-like dark petal bases and larger seeds that together characterize the hexaploid, hint that the combination AAGG × BB might be the better candidate. The matter is complicated by the fact that while both of the relevant diploids occur in the geographical vicinity of the two known occurrences of *Kosteletzkya racemosa* in southern Congo-Kinshasa and northwestern Zambia, none of the tetraploids are currently known to do so.

The preceding discussion is not meant to imply that *Kosteletzkya adoensis*, *Kosteletzkya buettneri* and *Kosteletzkya grantii* are the direct, immediate sources of the genomes A, B and G that are found in the polyploids, but rather that the lineages that gave rise to these three modern diploids also contributed their genomes relatively recently to the polyploids. The two interspecific hybrids that are the diploid counterparts, AB and AG, of the natural tetraploids whose genomic makeups are known, i.e. *Kosteletzkya borkouana* (AABB) and *Kosteletzkya begoniifolia*/*rotundalata* (AAGG), are only somewhat similar morphologically, not identical, to these tetraploids. Likewise the artificial tetraploid that is the full genomic counterpart of the wild species *Kosteletzkya begoniifolia* is similar to the latter, but distinguishable from it. Finally, both the triploid combination *Kosteletzkya begoniifolia*-*Kosteletzkya buettneri* (ABG), and the triploid combination *Kosteletzkya borkouana*-*Kosteletzkya grantii* (also ABG), look similar, but not identical, to the hexaploid *Kosteletzkya racemosa* (AABBGG).

### Kosteletzkya semota

*Kosteletzkya semota*, the third genomically distinctive tetraploid (recall that *Kosteletzkya rotundalata* and *Kosteletzkya begoniifolia* are genomically alike), appears to show little affinity with any of the three known diploid genomes. In effect the hexaploid *Kosteletzkya racemosa* offered all three known genomes to *Kosteletzkya semota* in a cross, but the 37-38 chromosomes of the latter could only recognize, on average, 6.6 *Kosteletzkya racemosa* chromosomes—the equivalent of about one-third of a genome. There were similar outcomes when *semota* participated in crosses with other tetraploids discussed above, yielding bivalent-equivalents ranging from 3.5 to 11.3, indicating at best a low-to-modest level of chromosomal homology with any of the other known genomes. Interestingly however, *Kosteletzkya semota* was shown to share over two-thirds of a genome (13.1 pairs) in a cross directly with the diploid *Kosteletzkya grantii*. These varied results leave uncertainties about the evolutionary position *Kosteletzkya semota*, so it seems best to give it a provisional designation of XXYY, which recognizes the species as distinctive and assumes an allopolyploid origin. At least one of its diploid progenitors, and its constituent genome, remains undiscovered or, more likely, extinct.

### Bivalent-equivalents

The decision to convert trivalents and quadrivalents to bivalent-equivalents (see Materials and methods) was intended to make Table 3 easier to read and interpret, but it is worth considering whether this conversion might have led to bias. In polyploid hybrids, multivalent associations sometimes indicate pairing within genomes (autosyndesis), which in turn suggests autopolyploidy—an interpretation that conflicts with the hypothesis of allopolyploidy that I have posited here. In the present case however, such an explanation is highly unlikely. Of the 618 meiotic cells examined, only nine had a single trivalent and only 18 others had a single quadrivalent. Moreover, 24 of these 27 multivalents occurred in diploid-diploid hybrids, and therefore could not be attributed, by definition, to autosyndesis. The three exceptions, involving one trivalent and two quadrivalents, were all found in hybrids in which the artificial tetraploid was one parent, and since I have shown here that this plant was derived from an interspecific diploid-diploid hybrid, its multivalents cannot be interpreted as indicating autopolyploidy.

### A hypothesized evolutionary (geographic and genomic) history of *Kosteletzkya*

Figure 6 depicts a reconstruction of the postulated genomic-phytogeographic history of the genus *Kosteletzkya* based on the cytogenetic evidence presented here. It assumes that the degree of chromosome pairing in the experimental hybrids can be used as a rough relative measure of the degree of evolutionary divergence of the parents of a cross. In support of this assumption, I note that in the well-studied malvaceous genus *Gossypium*, the African genomes A, B and E show pairing relationships among themselves (data from Konan et al. 2009) that are similar to those among the A, B, and G genomes of *Kosteletzkya*, andin the case of *Gossypium* the extent of the evolutionary divergence suggested by the degree of chromosome pairing is supported by both morphological evidence (Fryxell 1971) and molecular evidence (Cronn and Wendel 2004).

The reconstruction shown here indicates the reticulate nature of the evolution of *Kosteletzkya* in Africa, and also emphasizes that all of the early events in the history of the genus took place on the African continent. The lineage giving rise eventually to the B and G genomes is shown as separating from the A-genome lineage early in the evolution of the genus. This B-G branch itself branched in a more recent step, leading eventually to the extant African diploids *Kosteletzkya buettneri* and *Kosteletzkya grantii* respectively, while the A genome eventually gave rise to the extant diploid *Kosteletzkya adoensis*. The rest of the African diversification occurred at the polyploid level, and initially involved two separate interspecific hybridizations, each of which involved an A-genome plant—one in combination with a B-genome plant and the other with a G-genome plant. Following a doubling of the chromosome complements in these two hybrids, and subsequent evolution at the tetraploid level, the two resulted in three extant species: *Kosteletzkya borkouana* with genomic makeup AABB, and *Kosteletzkya begoniifolia* and *Kosteletzkya rotundalata*, each with AAGG. A cross between one of these tetraploids and a diploid bearing the third genome, followed by doubling, produced the hexaploid *Kosteletzkya racemosa* having a genomic makeup of AABBGG. Of the two possible hybrid combinations that might have led to this hexaploid, I have illustrated the one in which the tetraploid partner is from the *Kosteletzkya begoniifolia*-*Kosteletzkya rotundalata* (AAGG) lineage, since this alternative seems better supported by morphological evidence. The two postulated early-diverging genomes that led to the formation of the tetraploid *Kosteletzkya semota* have been designated here as XXYY, but in the depiction in Fig. 6, one of its genomes is tentatively shown as having its origin in the *Kosteletzkya grantii* lineage, since the cross *Kosteletzkya semota* × *Kosteletzkya grantii* yielded 13.1 chromosome pairs, the equivalent of more than 2/3 of a genome.

The atypically high level of fruit-set—63 percent—in the African tetraploid-tetraploid cross *Kosteletzkya begoniifolia* × *Kosteletzkya rotundalata* contrasts dramatically with the zero percent seen in all twelve of the other African hybrids for which there are fruit-set data (Table 3). This and the pairing evidence imply that the two parents diverged relatively recently from a common tetraploid ancestor, and this, too, is suggested in Fig. 6.

Finally, the history of *Kosteletzkya* in the New World was set into motion by a relatively recent dispersal to the New World of a B-genome-bearing *Kosteletzkya buettneri* ancestor, followed by a rapid radiation to yield the seven known diploids in that hemisphere. A similar pattern, in which one among several African genomes is also found in the New-World, can be seen in *Gossypium* (Endrizzi et al. 1985, Wendel and Cronn 2003) and in *Hibiscus* sect. *Furcaria* (see Menzel et al. 1983), and in both cases trans-Atlantic dispersals to the New World have been invoked**.**

In reporting on chromosome numbers in *Kosteletzkya*, I used the numbers evidence to suggest that Africa was the birthplace of the genus (Blanchard 2012). The data newly presented here add strength to this contention. The genomic and ploidy profiles of the African half of the genus are so strikingly more deep and complex than in the New World half as to lead almost inevitably to the view that, despite similar levels of morphological diversity, the New World taxa are of a much more recent origin.

Within the diversity of the New-World *Kosteletzkya* species there is little likelihood that any as-yet-undiscovered polyploids exist. Any interspecific New-World hybrid that formed would contain two B genomes, and in the event of a chromosome doubling, there would be four closely similar sets of chromosomes entering prophase I of meiosis. The result, barring strong preferential pairing, would be multivalent associations, confused and uneven segregation at anaphase, and consequently much reduced fertility**.**

An extension of this idea may explain why genome A, rather than one of the other two African genomes, is the one that is shared among the tetraploids *Kosteletzkya begoniifolia*, *Kosteletzkya borkouana* and *Kosteletzkya rotundalata*. While there is some disagreement about whether or not allopolyploidy occurs more commonly in hybrids between parents with a greater genetic distance between them (Chapman and Burke 2007, Buggs et al. 2008, Paun et al. 2009, Buggs et al. 2011), the situation in *Kosteletzkya* suggests that distance may count. Of the three pairwise combinations among the African diploids, only the two combinations AB and AG, have truly low levels of chromosome pairing—averaging only about 2 or 3 bivalents out of a potential 19. It is these two combinations that have been shown here to have given rise to the tetraploids *Kosteletzkya borkouana* and *Kosteletzkya begoniifolia*/*Kosteletzkya rotundalata* respectively. The third combination, BG, averages 9.1 bivalents—the equivalent of nearly half a genome. If a hybrid of the latter were to have formed and experienced a doubling of its chromosomes it would be much more likely than the other two to suffer serious meiotic problems. There is no present-day evidence of such a polyploid, and its existence would not be expected.

### Long-distance dispersal

The suggestion that a *Kosteletzkya* carrying a B genome made its pre-Columbian way across the Atlantic calls for an evaluation of the dispersal capabilities of the group. Stephens (1966) discussed the matter for *Gossypium*, which presents a similar problem of an amphi-Atlantic distribution of its A genome. Hochreutiner, in his revision of the genus *Hibiscus* (1900), considered the winged fruits of species of *Hibiscus* sect.* Pterocarpus* Garcke, 1849 and found no dispersal function for the wings since the capsules of *Hibiscus* dehisce in place to release their seeds. Instead he suggested that the wings mimicked those of the fruits of an East-African *Pavonia* in which, however, the wing-bearing mericarps actually individually enclose the seeds and could therefore presumably act as windborne disseminules. He later elaborated further on winged fruits in the Malvoideae (1913). Mattei (1917) rejected Hochreutiner’s interpretation and also extended the discussion to *Kosteletzkya*, saying that in both *Hibiscus* sect. *Pterocarpus* and in *Kosteletzkya* the capsule-valves separate from the fruiting axis, so that it was possible that incompletely laterally disarticulated capsule-valve pairs could become windborne and carry seeds with them. Hochreutiner later (1924) conceded that his earlier comments about mimicry had been weak, but he maintained that in his experience all of the capsule-valves in these plants separated at the same time from the axis, not in groups. He proposed instead that the wings were organs for dehiscence of the capsule, not for seed dissemination, and cited other not really comparable examples from elsewhere in the Malvoideae.

My experience with *Kosteletzkya* indicates that both Mattei and Hochreutiner were correct. Adjacent capsule-valves in *Kosteletzkya* often do cohere in pairs or threes and separate together from the fruiting axis, carrying one or two seeds with them (Blanchard in Verdcourt and Mwachala 2009). This can easily be observed when one harvests seeds by manually stripping a fruiting branch of its mature capsules. Many capsules fall completely apart with this rough handling, but some do not. Equally importantly, I found that it was not uncommon, when working in the greenhouse or collecting the plants in the wild, to discover that single or coherent capsule-valves had attached to clothing. The fruits of nearly all *Kosteletzkya* have bristly, sometimes hooked hairs either covering the whole surface or confined to the valve margins. These certainly are responsible for the adhesion, and I have no doubt that they may cling to fur and feathers as well, and aid in the dispersal of the seeds. To the extent that the wings help the bristles to project from the general fruit surface and therefore to be better exposed to passers-by, the wings aid in dispersal, but hardly in the form of windborne “flight.”

*Kosteletzkya pentacarpos* has been shown (as *Kosteletzkya virginica* [Linnaeus, 1753] A. Gray, 1849) to have an air space within the seed that permits it to float (Poljakoff-Mayber et al. 1992), and the same species has been credited with considerable salt tolerance and a seed coat that remains impermeable to water for some time (Poljakoff-Mayber et al. 1994). These features may aid in salt-marsh-to-salt-marsh dispersal as they apparently do in *Hibiscus moscheutos* Linnaeus, 1753, with which *Kosteletzkya pentacarpos* often shares habitat (Kudoh et al. 2006), and it is possible that the same characteristics could enhance the prospects of a trans-Atlantic crossing.

Dispersal over considerable distances appears to have been accomplished by several *Kosteletzkya* species. Known contemporary distributions suggest that *Kosteletzkya adoensis* has jumped from the African mainland to Madagascar, which is a minimum of 800 miles from the nearest known mainland population. It also appears to have dispersed westward from its main center in East Africa to the mountains of Cameroon (1100 miles) and the mountains of Sierra Leone (a further 1350 miles), with no known occurrences of the species—and no montane habitats—in the intervening areas. *Kosteletzkya begoniifolia* has made a similar jump from montane East Africa to Cameroon (1050 miles), and *Kosteletzkya borkouana* has dispersed from eastern Democratic Republic of the Congo and East Africa 1300 miles across a considerable expanse of the Sahara to the Borkou region of northern Chad (Blanchard 2013). In the New World, *Kosteletzkya depressa* has spread to the Cayman Islands and throughout the Greater Antilles, presumably from a mainland source (see Howard 1973), and *Kosteletzkya pentacarpos* has spread from the United States to Cuba and Bermuda. Moreover, if the latter turns out not to have been transported to Eurasia by human agency, it must have made the trans-Atlantic trip by long-distance dispersal, as did the carrier of the ancestral B-genome, but in this case dispersing from west to east.

It is worth noting, however, that some of these dispersal feats may have been aided in Africa by paleoclimatic cycles that provided geographically more benign intervening conditions (Quézel 1978, de Menocal 2011). In the case of *Kosteletzkya borkouana*, for example, the Sahara Desert was apparently largely vegetated at times during the interval from 15000 to 5000 years ago (de Menocal et al. 2000), and may have afforded the plant an opportunity to disperse by much shorter hops to northern Chad from East Africa. In the case of the Caribbean-island immigrants, on the other hand, the time frame in which these plants could have dispersed without having undergone appreciable subsequent divergence would have been too recent to attribute their dispersal to the narrowed or bridged ocean gaps known to have occurred earlier in the Cenozoic, so some sort of island-hopping seems to be the only plausible explanation for the island distributions of *Kosteletzkya depressa* and *Kosteletzkya pentacarpos* (see Pindell 1994).

Two *Kosteletzkya* species were unavailable for inclusion in the hybridization experiments reported here: *Kosteletzkya thurberi* from northwestern Mexico and the rare *Kosteletzkya batacensis*, endemic to the Philippine island of Luzon.It can be reasonably predicted that *Kosteletzkya thurberi* is a diploid of genomic makeup BB like all other New-World *Kosteletzkya* species. *Kosteletzkya batacensis*, on the other hand, remains a complete mystery. On the basis of general morphology—particularly of the fruits—the plant seems to belong in *Kosteletzkya*, although it is unique in the genus in being an annual. However its restricted range and its remote geographical location in relation to the rest of the genus would make any further speculation on its relationships almost reckless. There was an active maritime trade between Manila and Mexico for centuries, but suggestions of a Mexican origin for the plant (Merrill 1912, 1918, Borssum-Waalkes 1966), although appealing, are not supported by what is known of the several extant Mexican taxa (Blanchard 2008).

## Conclusions

In the two centers of diversity of *Kosteletzkya*, Africa and the northern Neotropics, the constituent species occur in approximately equal numbers and display similar ranges of morphological diversity. The results of the present study suggest that this apparent symmetry hides profound underlying differences in the evolutionary histories of the two groups. Pairing relationships in interspecific hybrids appear to show that *Kosteletzkya* in Africa underwent an early diversification at the diploid level, followed by a rich and complex history of allopolyploidy. In dramatic contrast, *Kosteletzkya* made a late appearance in the New World, where it underwent a rapid diploid-level diversification. These observations, especially if also borne out by a molecular study currently under way, lend strong support to my earlier contention, based on chromosome-number differences, that Africa is the birthplace of the genus. This scenario necessitates a long-distance dispersal, and the fruit and seed adaptations in *Kosteletzkya*,as well as known within-hemisphere dispersals, suggest such a capability.These observations also lend further weight to similar dispersals proposed by other workers to explain distributions in other malvoid genera, including the precursors of the cultivated cottons.
